# Editorial Notes

**Published:** 1923-03

**Authors:** 


### Editorial Notes.

**Light Ether Anæsthesia.**

FROM 1896 onwards, while acting as resident anæsthetist at the Bristol Royal Infirmary, Flemming has systematically employed his open ether drop bottle method without chloroform.

In 1908, in his contribution at the meeting of the British Medical Association, London, Mr. A. L. Flemming showed that the "open-ether-light-anæsthesia" method sufficed to avoid cyanosis, and further exploded the fallacy that hemorrhage was more prone to occur in anæsthesia by ether as compared with chloroform. We welcome the short summary of Flemming's further fifteen years' experience in light ether anæsthesia.

To Bristol belongs the credit of the discovery of nitrous oxide gas anæsthesia in the year 1799, and although ether narcosis was the gift of America, we venture to claim for Bristol some credit for the early development of the open method and particularly what is fairly described as light ether anæsthesia. Prior to the early years of the present century, despite certain advantages over chloroform, ether anæsthesia almost connoted bronchial secretions with increased liability to hemorrhage, and perchance secondary pulmonary complications.

**Need of Opportunity for Research.**

THE Editor feels that in his personal trial of per-rectal anæsthesia with ether in saline solution in 1902 Flemming was only less unhappy than in his experimental subcutaneous injection of 5 per cent. ether in saline, for this, it appears, gave him so much pain that his thoughts turned to the question of intravenous ether. In 1903, on applying at the University College for facilities to experiment on intravenous ether anæsthesia, he was discouraged by the difficulty in obtaining the necessary licence for any animal experiment. Though this at any rate would have been painless, we feel he wisely abstained from the risk of euthanasia by further trial on his own body. A year later, when Flemming and other anæsthetists were invited by the Bristol Dogs' Home Committee to advise on their method of killing dogs by painless anæsthesia, he asked permission to try what the late Mr. E. H. E. Stack suggested, viz. the intratracheal method of anæsthesia on dogs already anæsthetised, and which were anyhow to be killed without coming round. This was refused, consequently it was left to others to experiment on man. Years later the intratracheal method, one of the great advances in anæsthesic technique, was introduced by a German colleague. We regret that neither intravenous ether nor intratracheal anæsthesia came to us from Bristol owing to lack of facilities for the necessary observations.

**Postgraduate Activity.**

THE postgraduate courses in medicine arranged by the University continue to attract widespread approbation. *The Morning Post* for March 10th, 1923, contained the following paragraphs :—

"The classes of Medical Practitioners for whom the opportunity for increasing and revising their knowledge is especially needed to include officers on study leave, etc., but most importantly for General Practitioners who desire to revise from time to time their methods and conceptions in accordance with developments since the science of their student days.

"EXAMPLE OF BRISTOL.

"There has been published this week in the medical journals the syllabus of a course which is available in connection with the University of Bristol, and which might serve as a model for all postgraduate schemes where the particular objective is to arrange revision classes for General Practitioners. In connection with certain postgraduate instruction, arranged from time to time at localities in the neighbourhood of Bristol, a classified list of demonstrations is published which are available for selection by the medical men of the neighbourhood. The syllabus covers, to speak generally, the whole of therapeutics, and the postgraduate students can choose the particular course, general and special, which suits their needs."

A course of eight weekly lectures is now proceeding at the University, and the Director of Postgraduate Studies will gladly supply any of our readers with full particulars. In addition, ample opportunities are available for those who wish to carry out clinical work in Surgery, Medicine, and the special subjects.

